# Development and Validation of a Predictive Model for 6-Month All-Cause Mortality in Pulmonary Embolism

**DOI:** 10.3390/jcm15197357

**Published:** 2026-09-22

**Authors:** Zhiye Wu, Xuemei Wu, Xinhuang Hou, Weizhi Chen, Jun Lin, Qiaoyi Wu

**Affiliations:** 1 Trauma Center and Emergency Surgery Department, The First Affiliated Hospital, Fujian Medical University, Fuzhou 350005, China; wzy996999@163.com (Z.W.); cwz981159207@fjmu.edu.cn (W.C.); linjuniyeir@fjmu.edu.cn (J.L.); 2 Trauma Center and Emergency Surgery Department, National Regional Medical Center, Binhai Campus of The First Affiliated Hospital, Fujian Medical University, Fuzhou 350212, China; 3 Department of Hepatology, The First Affiliated Hospital, Fujian Medical University, Fuzhou 350005, China; 4 Department of Hepatology, National Regional Medical Center, Binhai Campus of The First Affiliated Hospital, Fujian Medical University, Fuzhou 350212, China; 5 Department of Vascular Surgery, The First Affiliated Hospital, Fujian Medical University, Fuzhou 350005, China

**Keywords:** pulmonary embolism, mortality, prognosis, nomograms, systemic inflammation response index, atrial fibrillation

## Abstract

**Background/Objectives**: Pulmonary embolism (PE) remains a major cause of cardiovascular mortality, yet existing risk stratification tools predominantly focus on short-term outcomes. In this study, we set out to develop and externally validate a predictive model for 6-month all-cause mortality in PE patients using routinely available clinical parameters. **Methods**: We conducted a retrospective analysis of patients with PE from two independent cohorts. The MIMIC-IV database served as the training set for variable selection using least absolute shrinkage and selection operator (LASSO) regression. External validation was performed using data from the First Affiliated Hospital of Fujian Medical University. Model performance was assessed through discrimination (area under the receiver operating characteristic curve [AUC]), calibration, and decision curve analysis. **Results**: The training cohort comprised 814 patients from the MIMIC-IV database, with a 6-month all-cause mortality rate of 21.3% (*n* = 174). LASSO regression initially selected 13 predictors. External validation in the local cohort (*n* = 217, 6-month all-cause mortality: 16.5%) refined these to four core predictors: respiratory rate, oxygen saturation (SpO_2_), and the systemic inflammation response index (SIRI). The parsimonious model demonstrated good discrimination (AUC = 0.81, 95% confidence interval [CI]: 0.74–0.88), excellent calibration (Hosmer–Lemeshow *p* = 0.367), and positive net clinical benefit across a range of threshold probabilities. After standardizing SIRI to absolute counts, SIRI was retained as a pre-specified inflammatory predictor. **Conclusions**: This simplified model incorporating four readily available clinical variables accurately predicts 6-month all-cause mortality in patients with PE.

## 1. Introduction

Pulmonary embolism (PE) represents a significant global health burden, with an estimated annual incidence ranging from 39 to 115 per 100,000 individuals [1,2,3,4]. Despite advances in diagnostic modalities and therapeutic interventions, PE-associated mortality remains substantial, particularly in the months following the index event [5,6,7,8]. Crucially, evidence suggests that the majority of PE-related deaths occur within the first six months, with the risk stabilizing thereafter; beyond this period, long-term mortality is primarily driven by underlying comorbidities rather than the index PE itself [4]. Accurate and timely risk stratification is therefore paramount for optimizing patient management.

Contemporary risk stratification in PE predominantly relies on tools such as the Pulmonary Embolism Severity Index and its simplified version [1,4,9]. While these scores demonstrate robust performance in predicting 30-day mortality, their utility in forecasting longer-term outcomes is less well established [10]. Furthermore, these instruments do not incorporate emerging prognostic indicators, such as markers of systemic inflammation, which may provide valuable insights into the underlying pathophysiology and disease trajectory.

A growing body of evidence has implicated systemic inflammation as a key determinant of adverse outcomes in various cardiovascular diseases, including venous thromboembolism [11,12]. Composite inflammatory indices derived from routine complete blood counts, including the neutrophil-to-lymphocyte ratio (NLR), platelet-to-lymphocyte ratio (PLR), and the systemic inflammation response index (SIRI), offer practical measures of the host inflammatory state [13,14,15,16,17]. However, the incremental prognostic value of SIRI relative to established clinical predictors in PE has not been rigorously evaluated in externally validated, multivariable models [15].

To address this gap, we conducted a dual-center retrospective study with the aim of identifying clinical predictors of 6-month all-cause mortality in patients with PE, using LASSO regression on a large, publicly available database (MIMIC-IV). Additionally, we sought to externally validate and refine these predictors in an independent Chinese cohort. To enhance model interpretability, we employed random forest and SHAP analyses. Finally, we aimed to develop a simple, clinically applicable nomogram for individualized risk assessment.

Although echocardiographic variables such as right ventricular dysfunction, tricuspid annular plane systolic excursion (TAPSE), and the right ventricular/left ventricular diameter ratio can improve PE risk stratification, they are not uniformly available [18,19]. Their absence in the present study is an acknowledged gap that may constrain model performance and is discussed in the Discussion Section.

## 2. Materials and Methods

### 2.1. Study Design and Data Sources

This study was a retrospective prediction model study based on two independent cohorts. The training set data were obtained from the MIMIC-IV database in the United States. The local cohort data were derived from the electronic medical record system of the First Affiliated Hospital of Fujian Medical University, collected from January 2015 to December 2022. This study adhered to the Declaration of Helsinki. Due to the retrospective and anonymized nature of the data, the use of the MIMIC-IV database was approved by the Institutional Review Board, and the use of local data was approved by the Ethics Committee of our hospital with a waiver of informed consent.

### 2.2. Study Population

Inclusion criteria: (1) patients diagnosed with PE using lung computed tomography pulmonary angiography (CTPA); (2) age ≥ 18 years; (3) complete hospitalization records. Exclusion criteria: (1) non-first admission; (2) length of hospital stay < 24 h; (3) severe hepatic or renal dysfunction, defined as alanine aminotransferase (ALT) or aspartate aminotransferase (AST) > 5 times the upper limit of normal, or estimated glomerular filtration rate (eGFR) < 15 mL/min/1.73 m^2^; (4) insufficient follow-up data.

### 2.3. Outcome

The primary outcome was all-cause mortality within 6 months after admission. In both cohorts, survival status was determined through hospital medical records, outpatient follow-up records, or telephone follow-up, and no deaths were excluded on the basis of timing relative to treatment. Time to death was defined as the number of days from the admission date to the date of death. Because cause-of-death data were not consistently available in either cohort, all deaths were treated as all-cause events.

### 2.4. Data Collection and Variable Definitions

Demographics and Clinical Characteristics were extracted from electronic medical records. These included age, sex, body mass index (BMI), length of stay (LOS), temperature (T), systolic blood pressure (SBP), diastolic blood pressure (DBP), heart rate (HR), respiratory rate (RR), and had lower SBP, DBP, and (SpO_2_).

Comorbidities and Lifestyle Factors included smoking history, alcohol consumption, hypertension, syncope, liver dysfunction, renal dysfunction, ischemic stroke, congestive heart failure (CHF), malignancy, chronic lung disease, and atrial fibrillation (AF). All comorbidities were determined based on ICD codes or discharge diagnoses and were represented as binary variables.

Laboratory Variables were collected from the first laboratory tests performed within 24 h of admission, including white blood cell count (WBC), neutrophil count (NUE), lymphocyte count (LC), monocyte count (MONO), platelet count (PLT), C-reactive protein (CRP), and albumin (ALB). Based on these, the following composite inflammatory indices were calculated: NLR = NUE/LC; derived NLR (d-NLR) = NUE/(WBC − NUE); lymphocyte-to-monocyte ratio (LMR) = LC/MONO; platelet-to-lymphocyte ratio (PLR) = PLT/LC; systemic immune-inflammation index (SII) = PLT × NUE/LC; systemic inflammation response index (SIRI) = NUE × MONO/LC; and aggregate index of systemic inflammation (AISI) = NUE × MONO × PLT/LC. All counts were expressed in 10^9^/L, and SIRI was calculated with absolute counts in both cohorts. The time from PE symptom onset to blood sampling was not available and could not be adjusted for in the analysis.

### 2.5. Statistical Analysis

Continuous variables were presented as mean ± standard deviation (for normally distributed data) or median (interquartile range) (for non-normally distributed data). Group comparisons for continuous variables were performed using the independent samples *t*-test or Mann–Whitney U test, as appropriate. Categorical variables were expressed as frequencies and percentages and were compared using the chi-square test or Fisher’s exact test. A two-sided *p*-value < 0.05 was considered statistically significant. There were no missing values in the final analytic datasets.

To identify potential predictors of 6-month all-cause mortality from the MIMIC-IV database, LASSO regression was employed. LASSO adds an L1 penalty term to the loss function, which can shrink some regression coefficients to zero, thereby enabling automatic variable selection and model regularization. Importantly, LASSO selects predictors on the basis of predictive performance rather than individual *p*-values; retention in the final model therefore does not require statistical significance in the subsequent multivariable model. Candidate variables included the original set of 33 demographic, clinical, comorbidity, and inflammatory indices, with length of stay excluded from all predictive modeling. Continuous variables were standardized before modeling, and categorical variables were coded as 0/1. Ten-fold cross-validation was used to select the optimal penalty parameter lambda with the criterion of minimizing binomial deviance, and the final model was determined using lambda.1se. The variables selected in the MIMIC-IV cohort were then entered into a second LASSO in the local cohort. Because SIRI was a pre-specified core inflammatory biomarker in this study, it was added to the local LASSO candidate set in addition to the 13 MIMIC-selected variables. Each variable retained by the local LASSO was examined by univariate logistic regression, and variables with *p* < 0.05 were entered into multivariable logistic regression.

The variables selected by LASSO in the MIMIC-IV cohort, together with the pre-specified SIRI, were then incorporated into a second LASSO regression using the local data, and univariate logistic regression was performed for each locally selected variable. Variables with *p* < 0.05 in univariate analysis were entered into multivariable logistic regression. The apparent discrimination (AUC) of the initial candidate model was calculated, and internal validation was performed using bootstrap resampling (1000 repetitions). Bootstrap percentile confidence intervals were also calculated for the coefficients of the final model.

Two machine learning methods, random forest and SHAP, were used for variable importance assessment. A random forest classification model was built on the local data (ntree = 500; mtry = square root of the number of variables), and the mean decrease in Gini was used as the variable importance metric. The fastshap package was used to calculate SHAP values, and the mean absolute SHAP value was used to measure global importance. Concurrently, LASSO regression was re-run on the local dataset using the training-selected variables to verify the stability of the reduced variable set.

Based on the selected core variables, a logistic regression model was re-fitted on the local data. A nomogram was plotted to visualize the model. The AUC and its 95% confidence interval were calculated, calibration was assessed with a calibration plot and the Hosmer–Lemeshow test, and decision curve analysis (DCA) was performed. AUCs were compared with PESI, sPESI, BOVA, and FAST using the DeLong test. Continuous net reclassification improvement (NRI) and integrated discrimination improvement (IDI) were calculated for the final model relative to each score. In the MIMIC-IV cohort, sPESI was calculated from available components; full PESI could not be reconstructed because altered mental status was not extracted.

All statistical analyses were performed using R software (version 4.4.1; R Foundation for Statistical Computing, Vienna, Austria). The glmnet (version 4.1.10), randomForest (version 4.7.1.2), fastshap (version 0.1.1), and rms (version 8.1.1) packages were used for LASSO regression, random forest modelling, SHAP value calculation, and nomogram construction and calibration, respectively.

## 3. Results

We included 814 patients with pulmonary embolism who had complete data from the MIMIC-IV database. Among them, 154 patients died within 3 months (18.9%) and 174 patients died within 6 months (21.3%). After comparing clinical parameters between patients who died within 6 months and those who survived, patients who died were older, had lower SBP, DBP, and SpO_2_, and had higher respiratory rates. Among comorbidities, liver dysfunction, renal dysfunction, CHF, malignancy, and AF were more frequent in deceased patients. In-hospital treatments, including thrombolytic therapy, vasopressor therapy, mechanical ventilation, and cardiopulmonary resuscitation, are summarized in Table 1.

Through LASSO regression in a binomial logistic regression model, the optimal penalty parameter lambda was selected using 10-fold cross-validation. From 814 patients in the MIMIC-IV database, 13 predictors associated with 6-month all-cause mortality were identified, including age, temperature, respiratory rate, oxygen saturation, smoking, liver dysfunction, renal dysfunction, congestive heart failure, malignancy, AF, neutrophil count, lymphocyte count, and NLR (Figure 1). These variables were used for the second LASSO in the local cohort.

In the local dataset, a total of 217 patients with PE were included, among whom 36 patients (16.5%) died within 6 months. After comparing clinical parameters between patients who died within 6 months and those who survived, patients who died had higher HR and RR, lower SpO_2_, and a higher proportion of AF. In-hospital treatment frequencies are presented in Table 2. AF management strategies were as follows: all 16 patients with AF received anticoagulation therapy (DOACs: 12 patients, warfarin: 4 patients); rate control with beta-blockers was prescribed in 11 patients (68.8%); rhythm control with amiodarone was used in 4 patients (25.0%). During the 6-month follow-up, 6 patients (37.5%) experienced AF relapse. Detailed AF management data are provided in Supplementary Table S1.

From the MIMIC-IV cohort, LASSO regression identified 13 potential predictors of 6-month all-cause mortality. These variables, together with the pre-specified SIRI, were subsequently entered into a second LASSO regression using the local hospital data, which retained respiratory rate, SpO_2_, AF, and SIRI. The initial 14-variable candidate model demonstrated good discrimination with an AUC of 0.81, and bootstrap internal validation yielded an optimism-corrected AUC of 0.73 (Figure 2).

We employed random forest and SHAP analyses to assess variable importance across all 14 local candidate variables (13 MIMIC-selected variables plus the pre-specified SIRI). The random forest model and SHAP analysis both identified SIRI, SpO_2_, respiratory rate, and AF as important contributors (Figure 3). LASSO regression re-run on the local dataset retained respiratory rate, SpO_2_, AF, and SIRI as the final predictors (Figure 4).

Based on the four locally selected predictors, we constructed a multivariable logistic regression model to predict 6-month all-cause mortality in the local cohort (Table 3). A nomogram was developed to visualize the model (Figure 5). The model demonstrated good discrimination, with an apparent AUC of 0.81 (95% CI: 0.74–0.88; Figure 6A) and a bootstrap optimism-corrected AUC of 0.79 (1000 resamples). Calibration was excellent (Hosmer–Lemeshow *p* = 0.367; Figure 6B). DCA demonstrated a positive net benefit compared with treat-all and treat-none strategies (Figure 6C). Removing SIRI significantly reduced discrimination (AUC 0.77; DeLong *p* = 0.025), supporting its incremental value.

In the MIMIC-IV cohort, sPESI yielded an AUC of 0.68; full PESI could not be reconstructed because altered mental status was not available. Compared with the initial 14-variable candidate model (AUC = 0.81), the simplified four-variable model achieved a bootstrap optimism-corrected AUC of 0.79. The comparison with PESI, sPESI, BOVA, and FAST is presented in Table 4.

## 4. Discussion

In this dual-center retrospective prognostic study, we developed and externally validated a predictive model for 6-month all-cause mortality in patients with PE. The final model, comprising four readily available variables—respiratory rate, SpO_2_, AF, and SIRI—demonstrated good discrimination, calibration, and favorable clinical utility in an independent validation cohort. After standardizing SIRI to absolute counts and removing length of stay from all predictive modeling, SIRI remained a significant predictor when it was pre-specified in the local LASSO candidate set.

Systemic Inflammation as an Exploratory Candidate. SIRI reflects the systemic inflammatory response by combining neutrophil, monocyte, and lymphocyte counts [20,21,22]. After standardizing SIRI to absolute counts (NUE × MONO/LC) in both cohorts, SIRI was not among the variables selected by the MIMIC-IV LASSO under the lambda.1se criterion. Nevertheless, because SIRI has been independently associated with adverse outcomes in cardiovascular and thromboembolic disease and was a pre-specified inflammatory biomarker in our study protocol, we pre-specified it for inclusion in the local LASSO candidate set. This two-stage approach—using MIMIC-IV to identify clinical predictors while preserving a biologically plausible inflammatory marker—was designed to balance data-driven discovery with mechanistic prior knowledge. In the final local model, SIRI achieved OR = 1.12 (95% CI: 1.00–1.25, *p* = 0.047), and its removal reduced the AUC from 0.81 to 0.77 (DeLong *p* = 0.025), supporting its incremental prognostic value. These findings support a clinically meaningful role for SIRI, while acknowledging that it may partly reflect general comorbidity or frailty.

Clinical Predictors and Pathophysiological Plausibility. The inclusion of RR, SpO_2_, AF, and SIRI in the final model aligns with the evolving understanding of PE pathophysiology and prognosis. Tachypnea and hypoxemia at presentation reflect right ventricular dysfunction and ventilation-perfusion mismatch. AF is a known risk factor for PE and may also serve as a marker of underlying cardiac dysfunction [23,24,25]. SIRI integrates neutrophil, monocyte, and lymphocyte counts and reflects the systemic inflammatory response [20,26,27,28]. Data on AF subtypes (paroxysmal, persistent, or permanent) were not available in either cohort, which limited subtype-specific analysis.

Model Performance and Parsimony. The simplified four-variable model exhibited good discrimination, underscoring the principle of parsimony in predictive modeling. In a sensitivity analysis, adding in-hospital treatments (thrombolysis, vasopressors, mechanical ventilation, and cardiopulmonary resuscitation) increased the AUC to 0.94, but these post-admission variables are strongly associated with disease severity and are not intended for admission-based prediction; they were therefore not included in the primary model.

Comparison with Existing Risk Scores. When full PESI components were available in the local cohort, PESI demonstrated an AUC of 0.85, exceeding that of our four-variable model (AUC = 0.81; DeLong *p* = 0.288). The negative NRI and IDI relative to PESI indicate that our model does not replace full PESI where complete scoring is feasible. However, PESI was designed for 30-day mortality prediction and requires components such as ‘altered mental status’—which is inherently subjective and often missing in retrospective data. Our model, by contrast, is intended for 6-month risk assessment using only objectively measurable variables that are routinely captured at admission, making it a practical alternative in settings where PESI components are incomplete or where longer-term prognostic information is desired. Other PE-specific scores, including BOVA, FAST, HOPPE, and GRACE, have been proposed [29,30,31,32]; in our local cohort, BOVA showed an AUC of 0.81, whereas FAST performed less well (AUC 0.60). Direct comparisons are limited by different outcomes and timeframes.

Several limitations merit acknowledgment. First, the retrospective design introduces the potential for selection bias and unmeasured confounding. Second, all-cause mortality includes deaths from cancer, cardiac disease, and other causes; because cause-of-death data were unavailable, competing-risk analysis could not be performed, and SIRI may be a generic marker of comorbidity or frailty. Third, AF subtypes, PE presumed etiologies, CTPA embolic burden and location, symptom-to-blood-sampling time, and echocardiographic variables (right ventricular dysfunction, TAPSE, RV/LV ratio) were unavailable; these limitations constrain comparison with hemodynamic scores. Fourth, treatment data were reported and used in a sensitivity analysis but were not part of the primary admission-based model. Fifth, LOS was excluded from predictive models because it depends on the healthcare system, which may limit international transferability. Sixth, BMI differed between cohorts, and the potential interaction of ethnicity or BMI with inflammatory indices remains to be explored. Finally, full PESI could not be reconstructed in MIMIC-IV because altered mental status was not extracted; only sPESI was compared in that cohort. These limitations do not negate the primary conclusion that a simple four-variable model predicts 6-month all-cause mortality in hospital-based patients with PE.

## 5. Conclusions

In summary, we developed and externally validated a parsimonious predictive model for 6-month all-cause mortality in patients with PE, based on four readily available variables: respiratory rate, SpO_2_, AF, and SIRI. The model demonstrates good discrimination, calibration, and clinical utility. This simple, practical tool may facilitate early identification of high-risk patients and inform clinical decision-making.

## Figures and Tables

**Figure 1:**
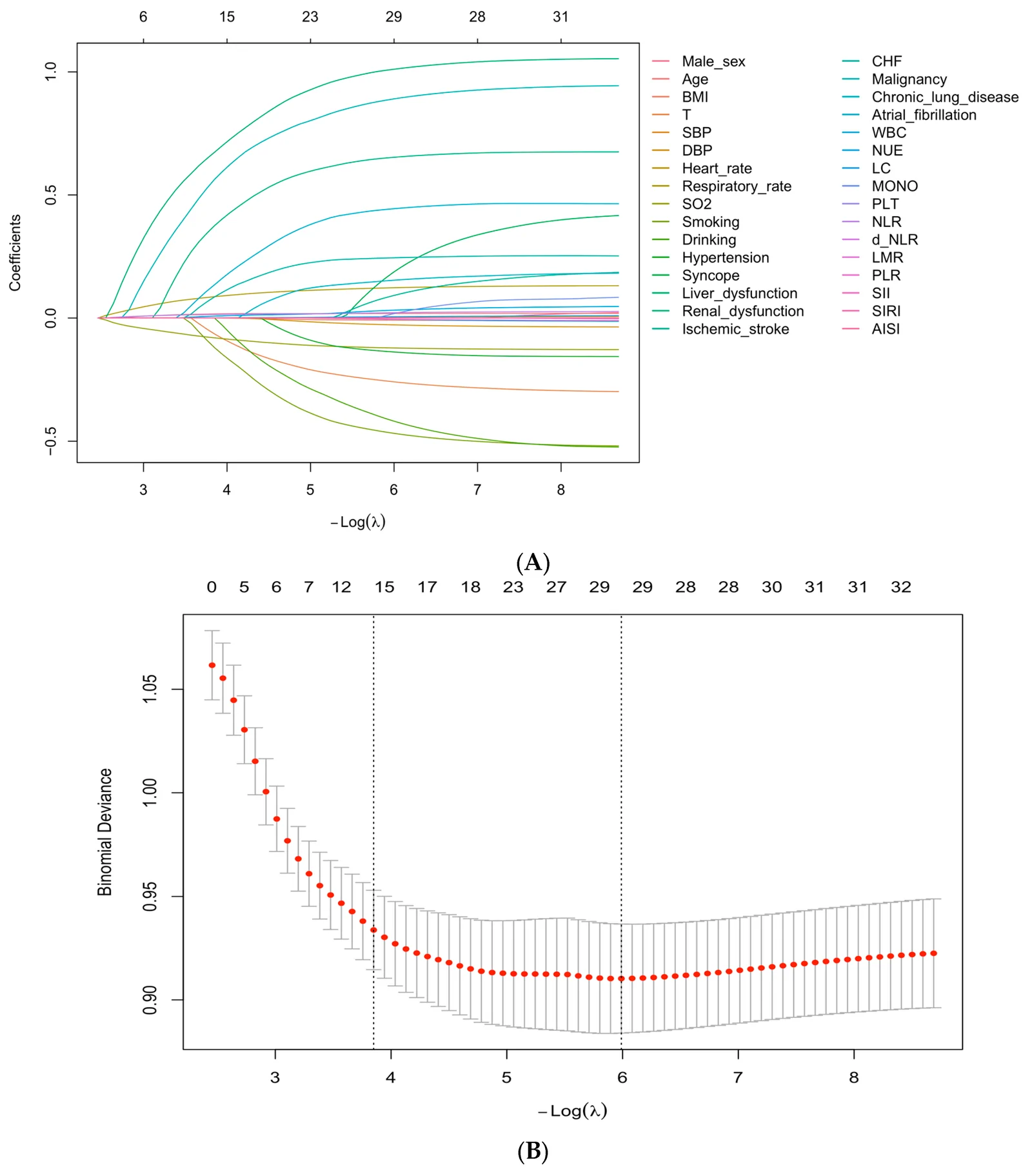
LASSO regression analysis of factors associated with 6-month all-cause mortality after pulmonary embolism in the MIMIC-IV database. (**A**) Coefficient profiles of the candidate variables included in the LASSO regression model. Each colored line represents the coefficient trajectory of one variable as the penalty parameter λ varies along the log scale. (**B**) Ten-fold cross-validated binomial deviance plotted against the log-transformed tuning parameter λ. Red dots represent the mean deviance at each λ, with grey error bars indicating ±1 standard error. The two vertical dotted lines indicate the optimal λ values selected by cross-validation: the left line marks λ.1se (the most parsimonious model within one standard error of the minimum deviance), and the right line marks λ.min (the minimum mean deviance). The 13 variables selected at λ.1se were identified as candidate predictors for the subsequent analysis.

**Figure 2:**
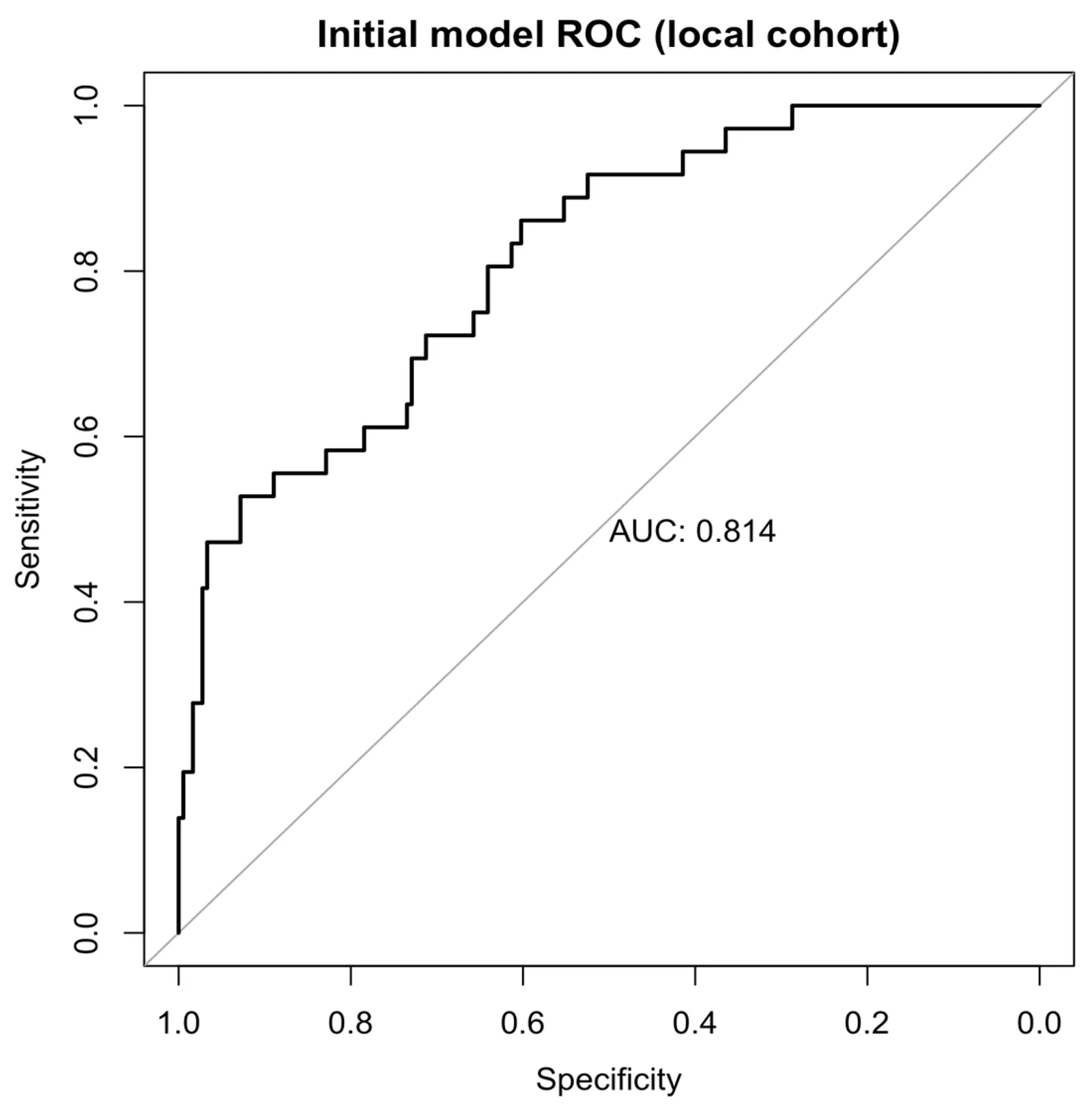
Receiver operating characteristic curve of the 14-variable candidate model in the local cohort, comprising the 13 MIMIC-selected variables and the pre-specified SIRI, along with the bootstrap optimism-corrected AUC.

**Figure 3:**
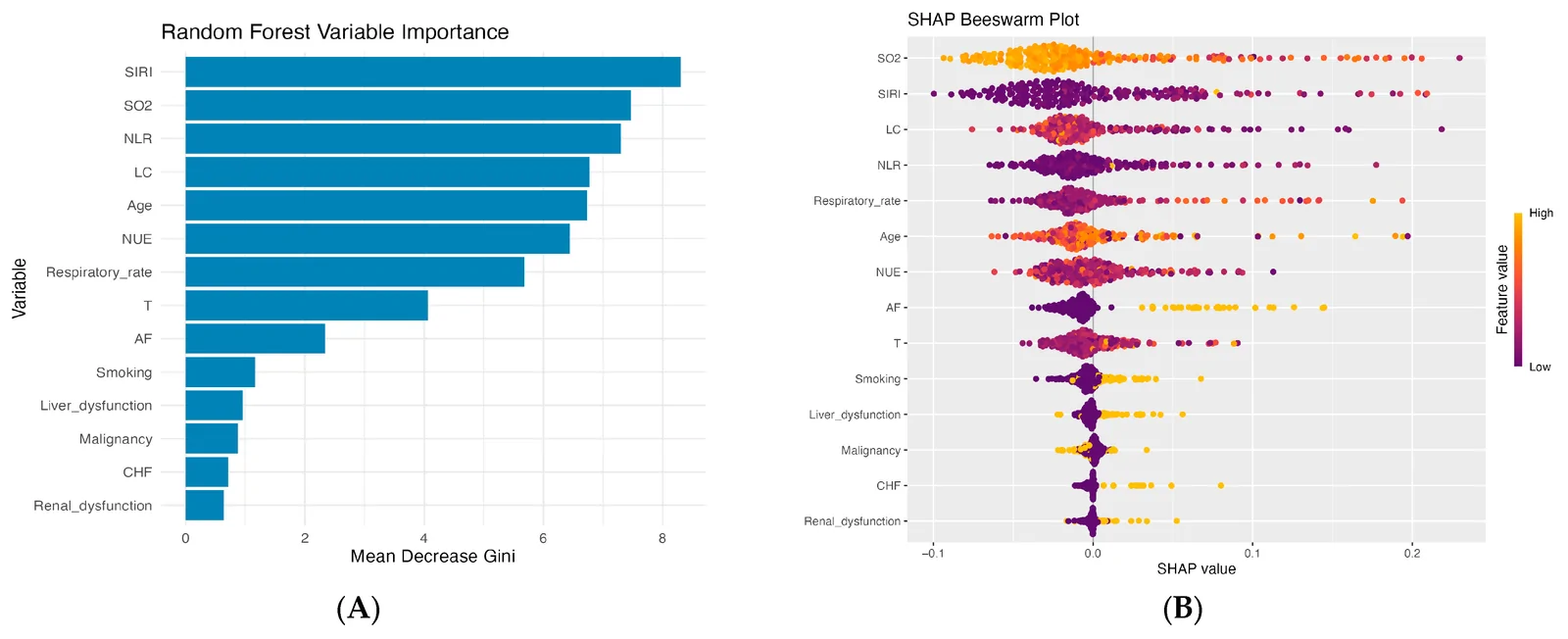
Feature importance analysis based on random forest and SHAP methods for the 14 local candidate variables (13 MIMIC-selected variables plus the pre-specified SIRI). (**A**) Variable importance from the random forest model, with features ranked by mean decrease in Gini. (**B**) SHAP beeswarm plot of variable importance.

**Figure 4:**
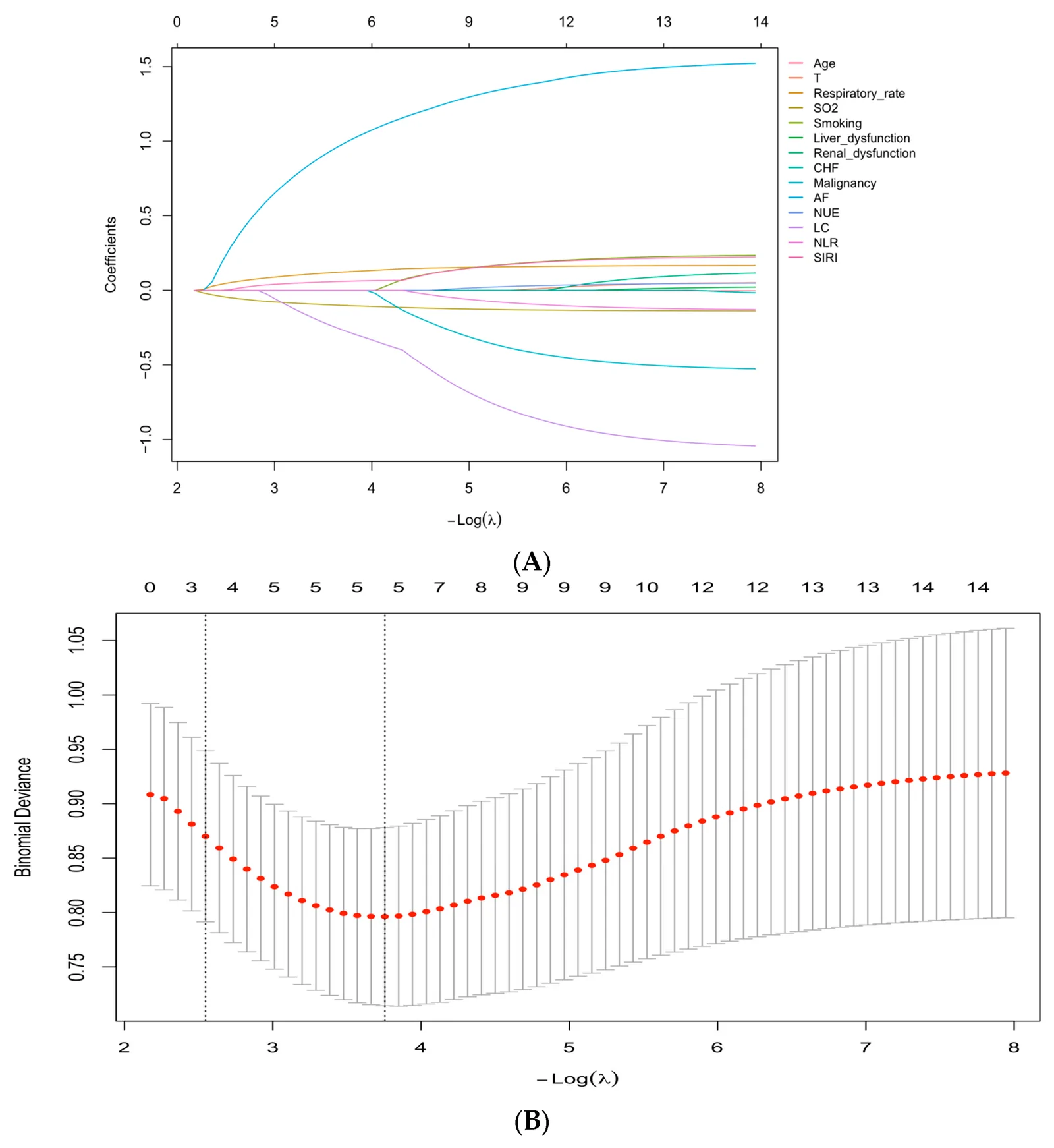
Second-stage LASSO regression for 6-month all-cause mortality in the local cohort. The 13 MIMIC-selected variables plus the pre-specified SIRI (14 candidate variables) were entered into the local LASSO model with 10-fold cross-validation. (**A**) Coefficient profiles against the log-transformed penalty parameter λ; the vertical dashed line marks λ.1se, at which respiratory rate, SpO_2_, AF and SIRI were retained. (**B**) Cross-validated binomial deviance against log(λ); points are mean deviances, error bars indicate ±1 standard error, and the dotted lines indicate λ. 1se (left) and λ. min (right). AF, atrial fibrillation; LASSO, least absolute shrinkage and selection operator; SIRI, systemic inflammation response index; SpO_2_, oxygen saturation.

**Figure 5:**
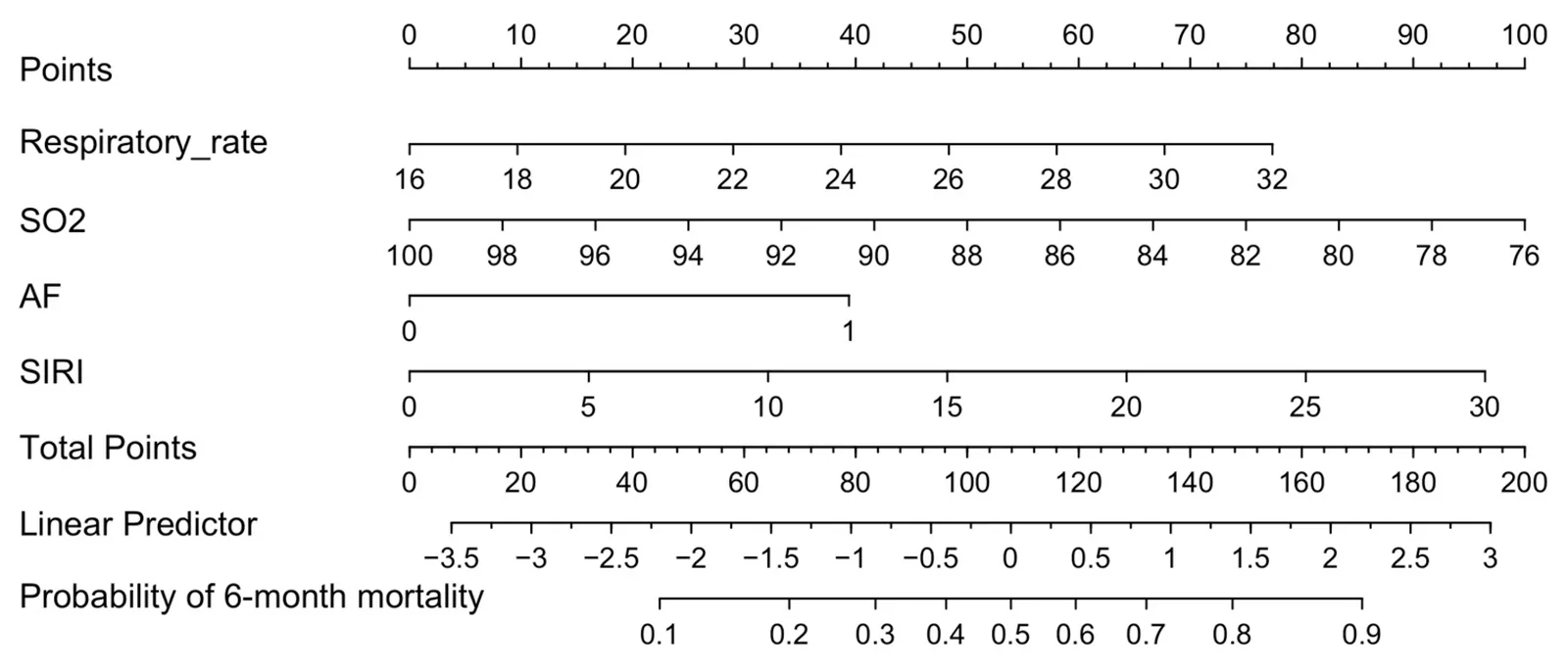
Nomogram for predicting 6-month all-cause mortality after pulmonary embolism in the local cohort. The total score corresponds to the probability at the bottom, summing each value of the variable.

**Figure 6:**
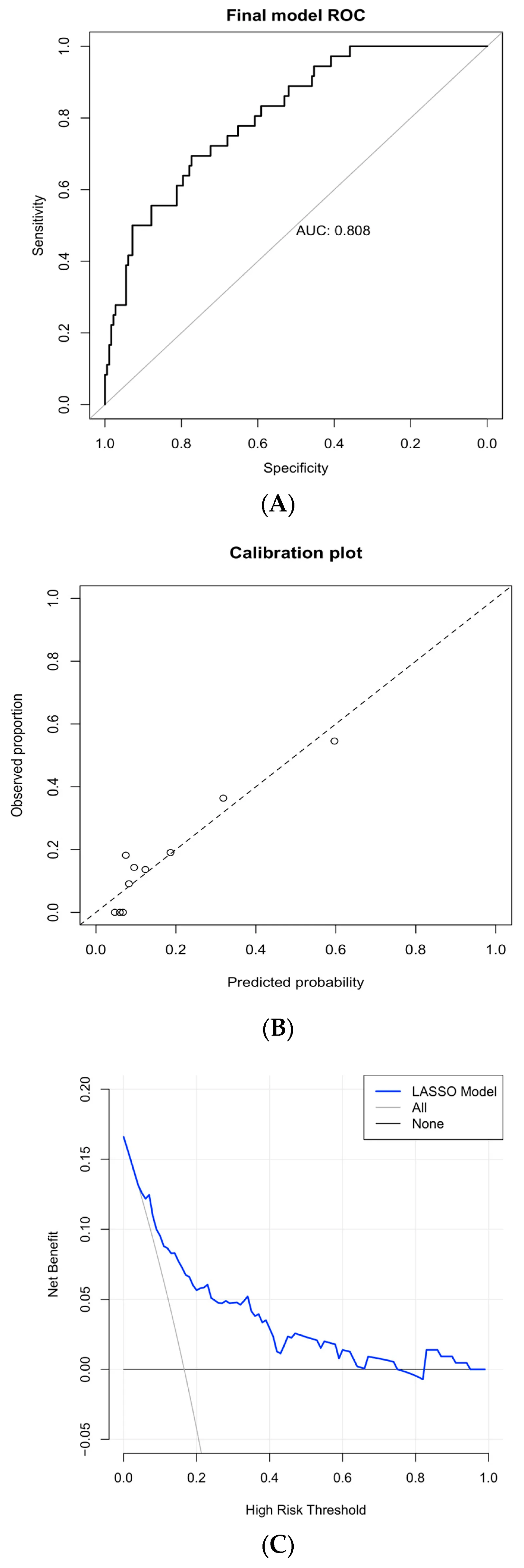
Evaluation of the predictive performance of the final model. (**A**) Receiver operating characteristic curve; (**B**) calibration plot; (**C**) decision curve analysis. The horizontal and vertical coordinates represent the threshold probability and net benefit, respectively.

**Table 1:** Comparison of Clinical Characteristics of Pulmonary Embolism Patients in the MIMIC-IV Database for Death and Survival within 6 Months.

Characteristic	Alive (*n* = 640)	Death (*n* = 174)	*p*-Value
Age (years)	62.00 (50.25, 72.00)	69.00 (57.75, 78.00)	<0.001 *
Sex *n* (%)			0.175
Male	346 (54.0%)	84 (48.3%)	
Female	294 (46.0%)	90 (51.7%)	
BMI (kg/m^2^)	28.23 (23.78, 34.09)	27.55 (23.53, 32.83)	0.215
T (°C)	37.00 (36.75, 37.33)	36.95 (36.66, 37.29)	0.100
SBP (mmHg)	118.82 (110.28, 130.16)	111.47 (102.65, 120.09)	<0.001 *
DBP (mmHg)	68.90 (61.84, 76.20)	63.51 (56.25, 71.44)	<0.001 *
HR (bpm)	90.71 (80.59, 99.55)	91.79 (83.07, 100.81)	0.062
RR (bpm)	20.60 (18.23, 23.20)	22.51 (20.36, 25.17)	<0.001 *
SpO_2_ (%)	96.63 (95.45, 97.73)	95.89 (94.05, 97.18)	<0.001 *
Smoking *n* (%)	240 (37.5%)	50 (28.7%)	0.032 *
Drinking *n* (%)	64 (10.0%)	9 (5.1%)	0.052
Hypertension *n* (%)	377 (58.9%)	105 (60.3%)	0.732
Syncope *n* (%)	6 (0.9%)	2 (1.1%)	0.682
Liver dysfunction *n* (%)	79 (12.3%)	51 (29.3%)	<0.001 *
Renal dysfunction *n* (%)	73 (11.4%)	35 (20.1%)	0.003 *
Ischemic stroke *n* (%)	52 (8.1%)	16 (9.1%)	0.644
CHF *n* (%)	156 (24.3%)	60 (34.4%)	0.007 *
Malignancy *n* (%)	132 (20.6%)	64 (36.8%)	<0.001 *
Chronic lung disease *n* (%)	139 (21.7%)	47 (27.0%)	0.140
Atrial fibrillation *n* (%)	104 (16.2%)	45 (25.8%)	0.004 *
WBC	12.20 (8.70, 15.80)	14.05 (9.00, 19.83)	0.006 *
NUE	9.27 (6.36, 12.66)	11.11 (7.13, 16.10)	0.001 *
LC	1.23 (0.77, 1.93)	0.93 (0.55, 1.48)	<0.001 *
MONO	0.69 (0.42, 1.04)	0.70 (0.40, 1.22)	0.446
PLT	217.00 (153.25, 295.00)	203.00 (119.50, 301.00)	0.162
NLR	7.12 (4.14, 12.23)	11.38 (6.59, 20.81)	<0.001 *
d-NLR	6.47 (3.77, 11.27)	10.60 (6.07, 19.51)	<0.001 *
LMR	1.97(1.04, 3.03)	1.36 (0.74, 2.15)	<0.001 *
PLR	18.76 (10.75, 38.61)	27.80 (13.11, 58.25)	<0.001*
SII	1434.79 (727.60, 3012.23)	2289.98 (1097.98, 4985.90)	<0.001 *
SIRI	4.75 (2.15, 9.74)	8.51 (3.42, 18.23)	<0.001 *
AISI	250.78 (107.93, 560.50)	373.11 (149.34, 1145.16)	<0.001 *
LOS (days)	17.00 (9.00, 28.00)	12.00 (6.00, 20.00)	<0.001 *
Thrombolytic therapy *n* (%)	210 (32.8%)	55 (31.6%)	0.834
Vasopressor therapy *n* (%)	365 (57.0%)	160 (92.0%)	<0.001 *
Mechanical ventilation *n* (%)	219 (34.2%)	109 (62.6%)	<0.001 *
Cardiopulmonary resuscitation *n* (%)	7 (1.1%)	8 (4.6%)	0.006 *

Abbreviations: AISI, aggregate index of systemic inflammation; BMI, body mass index; CHF, congestive heart failure; d-NLR, derived neutrophil-to-lymphocyte ratio; DBP, diastolic blood pressure; HR, heart rate; LC, lymphocyte count; LMR, lymphocyte-to-monocyte ratio; LOS, length of stay; MONO, monocyte count; NLR, neutrophil-to-lymphocyte ratio; NUE, neutrophil count; PLR, platelet-to-lymphocyte ratio; PLT, platelet count; RR, respiratory rate; SBP, systolic blood pressure; SII, systemic immune-inflammation index; SIRI, systemic inflammation response index; SpO_2_, oxygen saturation; WBC, white blood cell count. Continuous variables are presented as median (interquartile range) unless indicated otherwise; categorical variables are presented as *n* (%). * *p* < 0.05.

**Table 2:** Baseline Clinical Characteristics of Pulmonary Embolism Patients in the Local Cohort Stratified by 6-Month Mortality.

Characteristic	Alive (*n* = 181)	Death (*n* = 36)	*p*-Value
Age (years)	64.0 (49.5, 73.0)	67.5 (53.3, 75.0)	0.158
Sex			0.119
Female	106 (58.6%)	16 (44.4%)	
Male	75 (41.4%)	20 (55.6%)	
BMI (kg/m^2^)	23.6 ± 3.3	22.9 ± 3.1	0.233
T (°C)	36.5 (36.3, 36.7)	36.5 (36.3, 36.8)	0.461
SBP (mmHg)	126 (116.0, 137.5)	125.0 (107.8, 138.0)	0.223
DBP (mmHg)	76.0 (68.0, 84.0)	75.0 (69.3, 82.0)	0.533
HR (bpm)	76.0 (70.0, 80.5)	85 (75.3, 94.0)	<0.001 *
RR (bpm)	20.0 (19.0, 20.0)	20.0 (20.0, 24.0)	0.003 *
SpO_2_ (%)	97.0 (96.0, 98.0)	94.0 (90.3, 96.0)	<0.001 *
Smoking *n* (%)	44 (24.3%)	12 (33.3%)	0.258
Drinking *n* (%)	31 (17.1%)	4 (14.1%)	0.370
Hypertension *n* (%)	71 (32.9%)	20 (55.6%)	0.070
Syncope *n* (%)	22 (12.2%)	7 (19.4%)	0.365
Liver dysfunction *n* (%)	28 (15.5%)	10 (27.8%)	0.076
Renal dysfunction *n* (%)	16 (8.8%)	6 (16.7%)	0.263
Ischemic stroke *n* (%)	5 (2.8%)	17 (2.8%)	0.996
CHF *n* (%)	22 (12.2%)	7 (19.4%)	0.365
Malignancy *n* (%)	8 (4.4%)	5 (13.9%)	0.072
Chronic lung disease *n* (%)	40 (22.1%)	8 (22.2%)	0.987
Atrial fibrillation *n* (%)	8 (4.4%)	8 (22.2%)	<0.001 *
WBC	7.03 (5.83, 9.48)	8.4 (5.76, 10.47)	0.203
NUE	4.84 (3.74, 7.05)	6.03 (4.58, 8.23)	0.051
LC	1.43 (1.05, 1.99)	1.07 (0.67, 1.61)	0.002
MONO	0.41 (0.30, 0.54)	0.47 (0.30, 0.69)	0.089
PLT	226 (176.5, 287)	157 (133, 231.75)	<0.001 *
NLR	3.36 (2.22,5.60)	5.45 (2.99, 11.45)	<0.001 *
d-NLR	2.16 (1.55,3.49)	3.24 (2.04, 5.42)	<0.001 *
LMR	3.58 (2.39,5.00)	2.30 (1.33, 3.32)	<0.001 *
PLR	157.02 (105.71, 238.79)	178.35 (98.49, 269.24)	0.559
SII	770.43 (472.02, 1447.38)	1113.92 (445.86, 1978.62)	0.173
SIRI	1.35 (0.82, 2.49)	2.89 (1.25,5.35)	<0.001 *
AISI	319.84 (169.11, 642.16)	478.99 (175.33, 1295.38)	0.061
LOS (days)	10.0 (7.0, 16.0)	16.5 (11.5, 30.25)	<0.001 *
Thrombolytic therapy *n* (%)	64 (35.4%)	21 (58.3%)	0.017
Vasopressor therapy *n* (%)	11 (6.1%)	24 (66.7%)	<0.001 *
Mechanical ventilation *n* (%)	4 (2.2%)	18 (50.0%)	<0.001 *
Cardiopulmonary resuscitation *n* (%)	3 (1.7%)	11 (30.6%)	<0.001 *

Abbreviations: AISI, aggregate index of systemic inflammation; BMI, body mass index; CHF, congestive heart failure; d-NLR, derived neutrophil-to-lymphocyte ratio; DBP, diastolic blood pressure; HR, heart rate; LC, lymphocyte count; LMR, lymphocyte-to-monocyte ratio; LOS, length of stay; MONO, monocyte count; NLR, neutrophil-to-lymphocyte ratio; NUE, neutrophil count; PLR, platelet-to-lymphocyte ratio; PLT, platelet count; RR, respiratory rate; SBP, systolic blood pressure; SII, systemic immune-inflammation index; SIRI, systemic inflammation response index; SpO_2_, oxygen saturation; WBC, white blood cell count. Continuous variables are presented as median (interquartile range) unless indicated otherwise; categorical variables are presented as *n* (%). * *p* < 0.05.

**Table 3:** Univariate and Multivariate Logistic Regression Analyses Exploring 6-Month Mortality in Pulmonary Embolism Patients.

Characteristic	Univariate Logistic Regression		Multivariate Logistic Regression	
	OR (95% CI)	*p*-Value	OR (95% CI)	*p*-Value
RR (bpm)	1.33 (1.14, 1.54)	<0.001 *	1.18 (1.01, 1.39)	0.040 *
SpO_2_ (%)	0.85 (0.78, 0.92)	<0.001 *	0.86 (0.79, 0.94)	0.001 *
Atrial fibrillation	5.16 (2.18, 12.21)	<0.001 *	3.95 (1.47, 10.65)	0.007 *
SIRI	1.18 (1.05, 1.31)	0.004 *	1.12 (1.00, 1.25)	0.047 *

Abbreviations: RR, respiratory rate; SIRI, systemic inflammation response index. Continuous variables are presented as median (interquartile range) unless indicated otherwise. * *p* < 0.05.

**Table 4:** Comparison of the final model with PESI, sPESI, BOVA, and FAST in the local cohort.

Score	AUC	DeLong *p*	Continuous NRI	IDI
PESI	0.85	0.288	−0.47	−0.07
sPESI	0.68	0.002	0.06	0.14
BOVA	0.81	0.912	−0.40	−0.04
FAST	0.60	0.000	0.78	0.17

## Data Availability

De-identified data may be made available from the corresponding author on reasonable request, subject to institutional and ethics committee restrictions.

## Supplementary Materials

The following supporting information can be downloaded at: https://www.mdpi.com/article/10.3390/jcm15197357/s1, Table S1: Atrial Fibrillation (AF) Management Strategies in the Local Cohort (*n* = 16).
