# Supplementary material for: Development and Validation of a Predictive Model for 6-Month All-Cause Mortality in Pulmonary Embolism
Source: J Clin Med. 2026 Sep 22;15(19):7357. doi: 10.3390/jcm15197357 (PMC13665513; doi:10.3390/jcm15197357)
Supplement: Supplementary file 1 [file jcm-15-07357-s001.zip › jcm-4457903-supplementary.pdf]

Supplementary Table S1

Atrial Fibrillation (AF) Management Strategies in the Local Cohort (n = 16)

Category	Treatment / Outcome	Number of Patients (n)	Percentage (%)
Anticoagulation	DOACs	12	75.0
	Warfarin	4	25.0
Rate control	Beta-blockers	16	100.0
	Calcium channel blockers	11	68.8
Rhythm control	Amiodarone	3	18.8
	Catheter ablation	4	25.0
AF relapse	Relapse occurred	0	0
	No relapse	6	37.5

Note: DOACs, direct oral anticoagulants. Percentages are calculated based on the total number of AF patients (n = 16). Some patients received multiple rate-control or rhythm-control medications simultaneously.
